# Analyzing cross-college course enrollments via contextual graph mining

**DOI:** 10.1371/journal.pone.0188577

**Published:** 2017-11-29

**Authors:** Yongzhen Wang, Xiaozhong Liu, Yan Chen

**Affiliations:** 1 Transportation Management College, Dalian Maritime University, Dalian, Liaoning, China; 2 School of Informatics, Computing and Engineering, Indiana University Bloomington, Bloomington, Indiana, United States of America; East China Normal University, CHINA

## Abstract

The ability to predict what courses a student may enroll in the coming semester plays a pivotal role in the allocation of learning resources, which is a hot topic in the domain of educational data mining. In this study, we propose an innovative approach to characterize students’ cross-college course enrollments by leveraging a novel contextual graph. Specifically, different kinds of variables, such as students, courses, colleges and diplomas, as well as various types of variable relations, are utilized to depict the context of each variable, and then a representation learning algorithm *node2vec* is applied to extracting sophisticated graph-based features for the enrollment analysis. In this manner, the relations between any pair of variables can be measured quantitatively, which enables the variable type to transform from nominal to ratio. These graph-based features are examined by the *random forest* algorithm, and experiments on 24,663 students, 1,674 courses and 417,590 enrollment records demonstrate that the contextual graph can successfully improve analyzing the cross-college course enrollments, where three of the graph-based features have significantly stronger impacts on prediction accuracy than the others. Besides, the empirical results also indicate that the student’s course preference is the most important factor in predicting future course enrollments, which is consistent to the previous studies that acknowledge the course interest is a key point for course recommendations.

## Introduction

**Fig 1:**
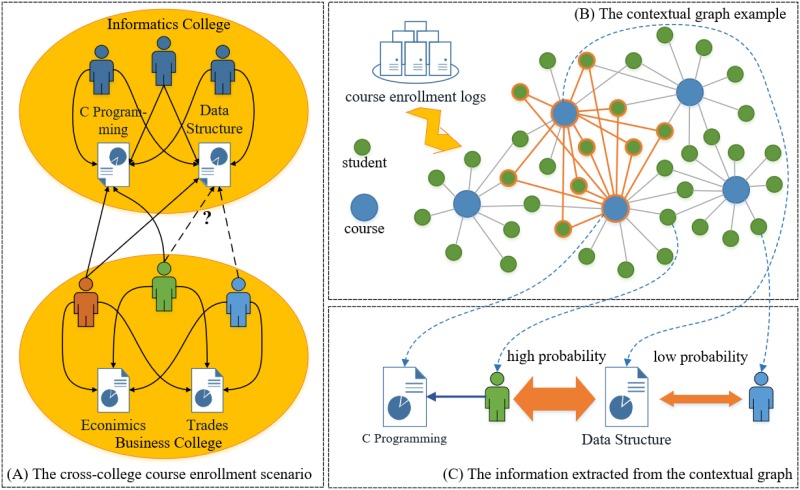
Framework of the proposed method. (A) The cross-college course enrollment scenario. The characters in navy color represent students in the Informatics College, whereas the characters in orange, green and blue s represent students in the Business College. Of the three art-major students, the orange studied both the *Data Structure* and *C Programming*, the green only enrolled in the *C Programming*, and the blue took none. (B) The contextual graph example. The two courses with thick edges represent the *Data Structure* and *C Programming* respectively, and the students with thick edges represent the ones who have studied both the two courses. (C) The information extracted from the contextual graph. As the green has taken the *C Programming*, he/she will have more and shorter connectivity paths to the *Data Structure* than the blue, which can be described as a higher probability.

In higher educational setting, the decision making during the course enrollment process prior to each semester is a key issue to successfully completing university degrees [1] and accomplishing career goals [2]. From eligible candidate courses, students would choose the ones that interest them as well as satisfy their degree and career development requirements. However, this process can be highly challenging, and usually depends on students’ own experiences. Without comprehensively considering the time, efforts and skills required by a course, locating right courses can be fairly difficult [3]. Moreover, as shown in Fig 1A, optimizing cross-college course enrollments would be the much more struggling scenarios. (In this paper, the “college” refers to a constituent part of a university, and generally “college”, “school”, “academy” etc are used interchangeably.) Since it is often less transparent for students to acquire an correct judgment on the courses outside their home colleges, they may lose enthusiasm to register the courses beyond their knowledge zones, even though some of such courses can be quite beneficial. Therefore, an intelligent program facilitating course enrollments is necessary in this circumstance. Meanwhile, from the university’s perspective, analyzing the cross-college course enrollments help not only to allocate teaching/learning resources efficiently, but also to build better learning experiences for students [4, 5].

During the past decades, educational data mining (EDM) has emerged as a paradigm towards designing models, tasks, methods and algorithms for exploring data in the context of education [6, 7]. Although there are many works in the literature illustrating the importance and potential of the EDM for analyzing/characterizing the course enrollment behaviors, to the best of our knowledge, few studies have investigated graph-based features to address the problems of this research. It is almost a common view in the previous studies to assume that explicit variables, such as students, courses and colleges, are mutually isolated in model space, which means that those potentially important relations among them are invariably ignored. Nonetheless, with remarkable advancements in graph mining [8], a growing number of scholars have exploited the variable relations to ameliorate their studies, ranging from learning user-item relatedness to improve item recommendations [9], to utilizing various structural relationships to enhance equipment-standard systems [10]. Thus, we have reasons to believe that these relations can be of great help to characterize the course enrollment process. And we have encapsulated all these latent variables in a contextual graph, then used a powerful representation learning algorithm to extract graph-based features for constructing our analysis models. Furthermore, in this study, we have also proposed a novel problem—the cross-college course enrollments. In an interdisciplinary environment of today, this problem can be rather more significant.

In a practical manner, it can be quite demanding for an art-major student who has not taken any computer-related courses to sign in *Data Structure*, but the one who has studied *C Programming* would have a greater chance to enroll the former. However, this kind of course information (e.g., course site and syllabus) is not always available as governmental and institutional policies impose strict regulations to ensure private and confidential [5]. Despite of that, these implicit information can be uncovered by the course enrollment logs plus some graph mining techniques too. For example, if we characterize students and courses as the nodes in a graph, as shown in Fig 1B, then the *C Programming* would have a high probability to **Random Walk** [11] to *Data Structure* through a large number of interconnected students, but such courses would have a much lower probability to walk to the art-major students outside the computer-related zones. That is to say, for the two students mentioned above, the one connected to the *C Programming* would have more connectivity paths (higher probability) to the *Data Structure* (see Fig 1C). More importantly, this contextual graph enables the implicit information in the course enrollment logs to turn intuitive and specific, and it is also convenient to supplement other kinds of variables (e.g., colleges and diplomas) so as to enhance its expression capability. Hence, we will characterize the course enrollment process via a contextual graph, and it has become an urgent task to extract useful features from the graph to improve analysis about this process.

**Fig 2:**
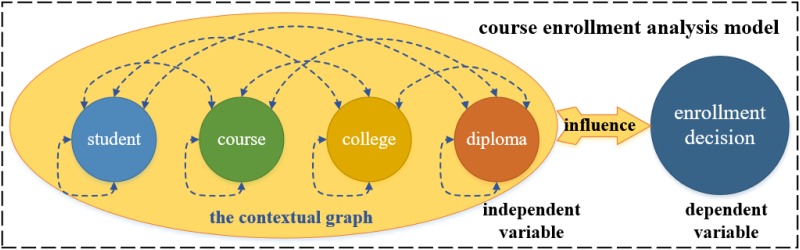
Illustration of the variable type transformation. The circles represent the nominal variables, and the blue dotted lines represent the ratio variables.

With feature engineering develops, current progress in representation learning for natural language processing has opened new ways for the feature learning of discrete variables [12, 13]. Recently, *Grover and Leskovec* put forward an algorithmic framework for learning continuous vector representations for nodes in graph [14], which formulates the feature learning in a graph as a maximum likelihood optimization problem. This technique aims to learn representations that embed nodes from the same graph community closely together, as well as the ones where nodes that share similar roles have similar embeddings. In this manner, we are able to represent all the nodes in a contextual graph so that various relations between any pair of nodes are measurable therefrom. In other words, it would enable us to transform the original nominal variables, i.e., the individual nodes in the contextual graph into the new ratio variables, i.e., the measured relations, as shown in Fig 2, which can be further utilized for deep knowledge extraction.

The goal of this study is to investigate whether the graph mining can be integrated into a course enrollment analysis framework, and we will explore the question, **What courses a student would enroll outside his/her home college in the coming semester?**, by leveraging the graph-based features. For our analysis models, the inputs can be divided into two groups, where one is originated from the course enrollment logs directly (the logs-based features), and another is extracted from the contextual graph (the graph-based features). Experiments on 24,663 students, 1,674 courses and 417,590 enrollment records demonstrate that, these graph-based features indeed contribute to ameliorating the enrollment forecasting, which indicates the feasibility of applying the graph mining to the EDM. To better verify the feature effects, we have also calculated the importance of all the features, and the related comparison shows that three of the graph-based features have significantly higher impacts than most of the others.

The main contributions of this paper are three-fold:

- It is an innovative attempt to introduce the graph mining into the EDM, which investigates students’ cross-college course enrollments via mining a contextual graph.
- Through exploring the heterogeneous relations among different kinds of variables in a deep learning framework, we extract graph-based features for analyzing the cross-college course enrollments.
- In more general, the proposed method can be used for the variable type transformation, such as from nominal to ratio.

## Literature review

The ability to predict what courses a student may enroll in the coming semester has significant quality assurance and economic imperatives [1, 5]. Specifically, the capability to determine course load and student interest in the future would offer an increased accuracy in the allocation of resources including the curriculum, learning supports and career counselling services. In the past years, a lot of studies have been done to illustrate the applications of data mining techniques in analyzing students’ behaviors on the course enrollments [6, 7]. Following is a brief description of some of the most relevant studies found in the literature.

One of the earliest applications of the EDM in predicting the course enrollments stems from *Luan* [15], which aims to infer the probability of transferring a student, and promote a timely intervention with students at a higher risk of leaving university. In this proposal, an *artificial neural network* has been employed, reaching an accuracy of 72%, as well as the *c5.0 rule induction*, gaining an accuracy of 80%. Based on that, universities can apply strategies to improve persistence and lessen dropout rate.

*Siraj and Abdoulha* have presented a two-step method to uncover the hidden information within universities’ enrollment data [16]. In this method, the *cluster analysis* is first performed to group the data into clusters according to similarity, and then the clustering results are used as targets for next prediction experiments. For the predictive analysis, three data mining techniques have been adopted, i.e., the *artificial neural network*, *logistic regression* and *decision tree*, and reach to an accuracy of more than 99%. Similarly, *Hsia et al.* have applied three data mining techniques successively to study course preference and course completion rate in the extension education courses [17]. Firstly, the *decision tree* is implemented to build up a tree relation, which is used to find the preferred courses. Next, the *link analysis* is utilized to discover the correlations between the preferred course category and the enrollee profession. Finally, the *decision forest* is adopted to find the preferred courses of enrollees from different sectors, along with the probability of course completion by sector.

Subsequently, *Nakhkob and Khademi* have intended to predict the rate of student enrollments in the coming years, where fifteen different *artificial neural networks* are constructed, and two ensemble methods, i.e., the *bagging* and *boosting* are utilized to increase accuracy [18]. Besides, three extra data mining techniques, including the *decision tree*, *naïve bayes* and *logistic regression* are implemented and evaluated, and the related comparison indicates that the *bagging* method is the most accurate one of all.

A recent study by *Gomes* has presented a predictive approach about how to support administrative necessities of a course director [19]. Three prediction topics have been analyzed, including the number of students per non-optional curricular unit, the number of students enrolling in optional curricular units, and the number of students per optional curricular unit. These topics are examined separately and different predictive models are formulated for each case, where all the corresponding models have proven to perform better than the naive estimates calculated from previous occurrences of curricular units or semesters and their averages.

From the perspective of course recommendations, *Vialardi et al.* have investigated the rationale behind design of a recommendation system in order to support the course enrollment process by means of students’ academic performances [3]. To build this system, the *c4.5*, *knn*, *naïve bayes*, *bagging* and *boosting*, five data mining techniques have been employed and compared, and the corresponding recommendations are only based on the academic performances of students. Then *Aher and Lobo* have shown how the combination of *cluster analysis* and *association rule* algorithm is helpful in course recommendation system, which recommends courses to students based on the choice of other students for a particular set of courses collected from the Moodle [20]. With experimental results, the combination of simple *k-means* and *apriori* could increase the strength of association rules, so this recommendation system would help students select proper course combinations according to their interest. Soon afterwards, *Aher* has put forward a better combination of *expectation maximization clustering* and *apriori*, and an open source data mining tool Weka is used to verify the results [21].

Notwithstanding the previous works in this field have clearly demonstrated the potential for data mining to provide course recommendations, there are still relevant factors being under-investigated, which could be utilized to further supplement these methods. *Kardan et al.* have attempted to identify latent factors that would affect students’ satisfaction on enrolled courses, and predicted the final number registrations in each course after the course enrollment process [22]. In this study, a neural network-based system has been implemented to simulate students’ behaviors on the enrollments for choosing eligible courses in an on-line university. Then *Ognjanovic et al.* have proposed a method to extract the student preference from resources available in the teaching manager information system [5]. And the extracted preference is analyzed through the *analytical hierarchy process*, a mature decision making technique for handling the multidimensional and sometimes conflicting preferences of individuals, which is further used to predict the course enrollments for students.

This study, unlike the prior works, presents a novel approach to characterize students’ behaviors on the cross-college course enrollments by leveraging a contextual graph. On this basis, various relations between different kinds of variables would become quantitatively measurable at the granularity of individuals, where each student, each course, each college and so forth are no longer isolated from one another. As discussed in the previous section, these relations encapsulate important implicit information about enrollment patterns. However, among all the reviewed works, few have taken this into account, and only measured them at the granularity of groups via the *cluster analysis* [16, 20, 21, 23]. Meanwhile, thanks to the excellent scalability of the contextual graph, this study has been launched on a big data environment with up to 417,590 enrollment records, which makes our results more convincing.

## Methodology

With regard to the cross-college course enrollments, in this study, the inputs are a student and a course outside his/her home college, and the output is whether he/she will enroll the course. Although many factors influence in the analysis accuracy, so far there is no standard way to select features for this prediction task. Limited by available data, nineteen features are studied in this paper, where thirteen of them are the logs-based features and the other six are the graph-based ones.

### Overview of the logs-based features

The logs-based features used in this study are listed in Table 1. To be clear, these features actually stem from the explicit nominal variables, and they are classified into six categories.

**Table 1:** Definitions of the logs-based features.

No	Feature Name	Description	Category
1	stuGdr	the gender of a student	demographics
2	stuAge	the age of a student	
3	stuLev	the educational level of a student	academic background
4	stuSub	the educational subject of a student	
5	stuGpaAve	the mean GPA of a student	academic performance
6	stuGpaStd	the standard error of GPA of a student	
7	stuRakInSch	the ranking of a student in his/her home college	
8	schNumCrsAve	the mean number of courses per student in his/her home college	academic requirement
9	schNumCrdAve	the mean number of credits per student in his/her home college	
10	crsGrdAve	the mean of a course’s grade	course difficulty
11	crsGrdStd	the standard error of a course’s grade	
12	crsNumStu	the number of students having enrolled a course before	course attraction
13	crsNumCrd	the number of credits gained after passing a course	

For the first two features, the previous studies have indicated an association between the demographic characteristics and the decisions concerning a student’s learning interest [24, 25]. Meanwhile, we have extracted another two background features, i.e., the educational level and subject of a student, which can be defined as his/her academic background. The third point about a student focuses on the academic performance, which describes his/her competence, based on the GPA he/she gained in the prior courses. Several works in the literature have reported that the academic performance is one of the key factors when recommending courses to students [3, 26]. Finally, we have tallied up the mean number of courses and credits per student in his/her home college, and used them to represent the academic requirement.

As for the characteristics of a course, *Babad* has pointed out that these attributes play a pivotal role in students’ choosing their courses [27]. *Greenwald and Gillmore* found that students prefer to enroll the courses that tend to give higher grades [28]. That is because, generally, the grades rather than the studying itself becomes the primary goal of students, and they may need decent grades to achieve the future admissions into advanced educations or well-paying jobs. Thus, we have included the mean and standard error of a course’s grade as two features into the inputs, and grouped them as the course difficulty category. Furthermore, we have also counted the number of students having enrolled a course before, and the number of credits gained after passing a course, which reflects a course’s attraction.

### Generating the course-enrollment contextual graph

In this section, we integrate all the isolated variables in a novel contextual graph, which enables the in-depth information extraction. We have used a directed heterogeneous graph *G* = (*V*, *E*) to embody the various organizational relations. In this graph, we have defined a node type mapping function *τ*: *V* → *O* and an edge type mapping function *ϕ*: *E* → *R*, where each node *v* ∈ *V* belongs to one particular variable *τ*(*v*) ∈ *O*, and each edge *e* ∈ *E* belongs to one particular relation *ϕ*(*e*) ∈ *R*. If two edges belong to the same relation, then they share the same starting variable as well as the same ending variable. The types of the nodes and the edges are presented in Table 2.

**Table 2:** Nodes and edges in the contextual graph.

	Type	Description
node	student	the student variable
	course	the course variable
	college	the college variable
	diploma	the diploma variable
edge	course → student	the course to student via the enrollment relation
	studnet → college	the student to college via the affiliation relation
	college → course	the college to course via the provider relation
	student → diploma	the student to diploma via the major relation
	student → student	the student to student via the upgrade relation

**Fig 3:**
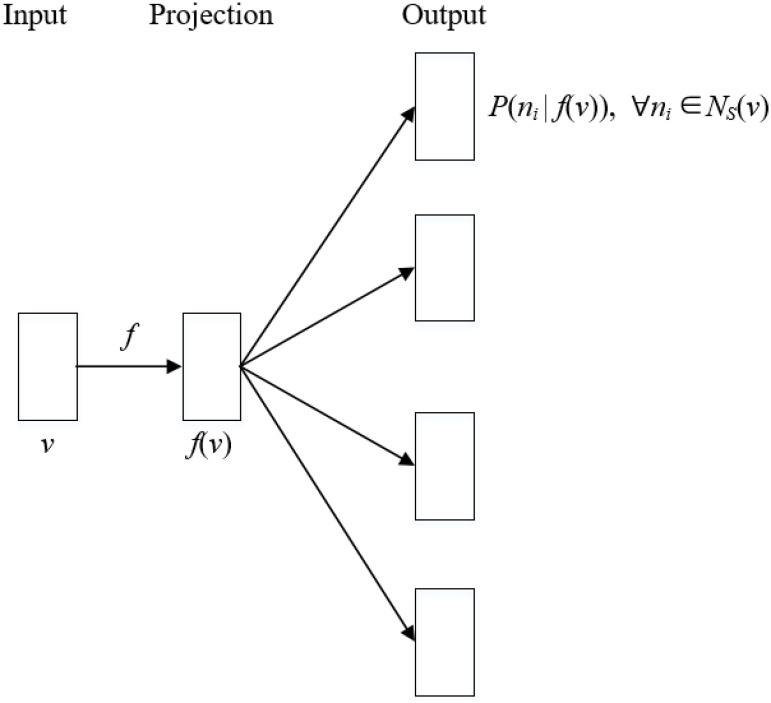
Illustration of the skip-gram network.

By leveraging this contextual graph, the implicit information hidden in the course enrollment logs would become intuitive and specific. For instance, it is feasible to identify the connections among courses, and which groups of students have the similar course preference. This deep information cannot be extracted from the logs directly, and it could be conducive to improving the cross-college course enrollment analysis. In this study, we have adopted the *node2vec* (the code can be found in S1 Code), a well-performing representation learning algorithm, to learn the continuous vector representations for nodes in the contextual graph [14]. The *node2vec* models the relations between a target node and its graph neighborhood through the *skip-gram* network, which is one of the *artificial neural network* architectures that are widely used in the natural language processing [13], as shown in Fig 3.

Let $f\in R^{|V|\times d}$ be the weight matrix from the input layer to the projection layer, where *f*(*v*) is the continuous vector representation of the target node *v*, and *d* is the parameter specifying the number of dimensions of the representations. For every node *v* ∈ *V*, we define *N*_*S*_(*v*) ∈ *V* as its graph neighborhood produced by a neighborhood sampling strategy *S*. The *node2vec* seeks to optimize the objective function (see Eq 1)

$$\begin{array}{c} \max_{f}\sum_{v\in V}\log P\left(N_{S}\left(v\right)|f\left(v\right)\right)=\max_{f}\sum_{v\in V}\sum_{n_{i}\in N_{S}\left(v\right)}\log(\frac{\exp(f\left(n_{i}\right)\cdot f\left(v\right))}{\sum_{u\in V}\exp\left(f\left(v\right)\cdot f\left(u\right)\right)}) \end{array}$$ (1)

using the *stochastic gradient descent* with the *negative sampling* [29], which maximizes the log-probability of observing a graph neighborhood *N*_*S*_(*v*) for a node *v* conditioned on its vector representation *f*(*v*). When the objective function is optimized, we would obtain a fine-tuned weight matrix *f* at the same time, i.e., the vector representations of all the nodes in the contextual graph.

In order to generate an suitable graph neighborhood *N*_*S*_(*v*) for a target node *v*, the *node2vec* employs a biased random walk procedure to sample the nodes that are in accordance with the neighborhood definition, as shown in Eq 2.

$$\begin{array}{c} P\left(l_{i}=x|l_{i-1}=u\right)=\{\begin{array}{cc} \frac{\pi_{ux}}{Z} & if\;(u,x)\in E\\ 0 & otherwise \end{array} \end{array}$$ (2)

**Fig 4:**
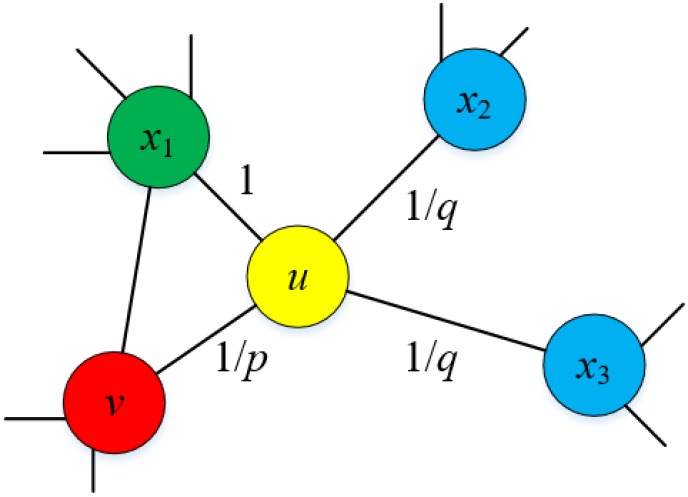
Illustration of the random walk procedure in node2vec. This random walk just traverses from *v* to *u* and now is evaluating its next move out of the node *u*. Edge label indicates the corresponding search biases *α*_*pq*_.

Here, we denote *l*_*i*_ as the *i*^*th*^ node in a random walk routine starting with *l*_0_, and *π*_*ux*_ as the unnormalized transition probability between the nodes *u*, *x* ∈ *V*, and *Z* as the corresponding normalizing constant. Consider a random walk that just goes through the edge (*v*, *u*) ∈ *E* and now stays at the node *u*, as shown in Fig 4. This walk now needs to determine the next move so it calculates the transition probability $\frac{\pi_{ux}}{Z}$ on all the edges leading from *u*. We set the unnormalized transition probability to *π*_*ux*_ = *α*_*pq*_(*v*, *x*) (see Eq 3),

$$\begin{array}{c} \alpha_{pq}\left(v,x\right)=\{\begin{array}{cc} \frac{1}{p} & if\;d_{vx}=0\\ 1 & if\;d_{vx}=1\\ \frac{1}{q} & if\;d_{vx}=2 \end{array} \end{array}$$ (3)

where the *d*_*vx*_ is regarded as the shortest path distance between two nodes *v* and *x*.

Notice that when conducting the biased random walk to generate a graph neighborhood, two parameters, i.e., a return parameter *p* and an in-out parameter *q* control how soon the walk explores and leaves the neighborhood of the starting node, which thereby reflects an affinity for different notions of the node equivalence (homophily and structural equivalence). Specifically, *p* controls the probability of immediately backtracking a node in the random walk, and *q* enables the walk to differentiate between the inward and outward nodes [14]. And it should be noted that the values of *p* and *q* heavily rely on application situations, and we have done an experiment to tune the parameters for our contextual graph. Through a flexible graph neighborhood definition and a biased random walk procedure, the *node2vec* is expressive enough to capture the diversity of connectivities observed in the contextual graph.

However, for our analysis task, what we really concerned about are the relations instead of the nodes, i.e., we are going to measure whether a kind of relation exists between a pair of nodes in the contextual graph. Therefore, we would need an operator defined for any pair of nodes even though the relation does not exist between the pair because this way makes the edge representations compatible to the link prediction. For two given nodes like *u* and *v*, an appropriate binary operator over the corresponding vectors *f*(*u*) and *f*(*v*) can generate an edge vector representation *g*(*u*, *v*) such that $g:V\times V\to R^{d}$. Table 3 summarizes four generally defined binary operators recommended in [14], and we would investigate their effects on our analysis models in the experiment section.

**Table 3:** Binary operators for learning the edge vector representations.

Operator	Symbol	Description
Average	⊕	$\left[f\left(u\right)⊕f\left(v\right)\right]=\frac{f(u)+f(v)}{2}$
Hadamard	⊙	[*f*(*u*) ⊙ *f*(*v*)] = *f*(*u*) * *f*(*v*)
Weight-1	|| ⋅ ||_1_	||*f*(*u*) ⋅ *f*(*v*)||_1_ = |*f*(*u*) − *f*(*v*)|
Weight-2	|| ⋅ ||_2_	||*f*(*u*) ⋅ *f*(*v*)||_2_ = |*f*(*u*) − *f*(*v*)|^2^

### Extracting the graph-based features

In this paper, the main task is to analyze students’ behaviors on the cross-college course enrollments. For the sake of making sense as well as ease of interpretation, six features are extracted from the contextual graph, and are classified into two categories, as listed in Table 4.

**Table 4:** Definitions of the graph-based features.

No	Feature Name	Description	Category
1	stuCrs	the distance from a student’s course centroid to a course	course preference
2	stuCrsInSch	the distance from a student’s course centroid to the course centroid in his/her home college	
3	stuCrsOutSch	the distance from a student’s course centroid to the course centroid in a course’s college	
4	crsStu	the distance from a course’s student centroid to a student	course appropriateness
5	crsStuInSch	the distance from a course’s student centroid to the student centroid in his/her home college	
6	crsStuOutSch	the distance from a course’s student centroid to the student centroid in the course’s college	

**Fig 5:**
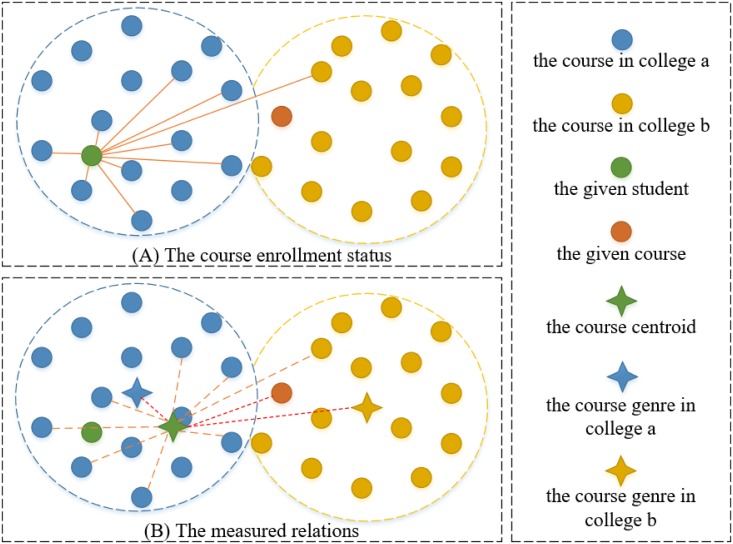
Illustration of the graph-based features. (A) The course enrollment status. (B) The measured relations.

Intuitively, a student’s interest in a course plays a crucial role in the enrollment decisions. During the course enrollment period, a student often selects the most desirable courses among the alternatives on the basis of available information [30, 31]. The interest is a latent variable, which, in prior studies, can be explored by interviews and questionnaires with a high cost. In this study, by mining a large course enrollment context graph, a student’s interest can be represented by the centroid of courses that he/she has already taken, and the distance between the centroid and a candidate course can be important to characterize the likelihood that the student will take this course. (The vector representations for centroids can be easily calculated by averaging the related nodes’ vectors [32].) As Fig 5 shows, the shorter the distance, the greater chance that the student will be interested in (taking) the course. Note that, when inferring whether a student will enroll a given course, the candidate course needs to be excluded from his/her course centroid in order to avoid bias. In addition, we have also calculated the course centroid per college, and defined that as the corresponding course genre. Thus, for a student and a course outside his/her home college, we can figure out two different course genres, and measure the distances from his/her course centroid to the two respectively. In this way, we can estimate the student’s interest in courses within or outside his/her home college.

As another factor, it can be fairly important to characterize a student’s eligibility when choosing a course [33]. In this study, we use the centroid of all the students (nodes) who took the target course to estimate the average knowledge requirement. Then, the distance from the student centroid to a given student can be used to characterize the student’s eligibility for taking this course. When a student is close to a course’s student centroid, e.g., a student from *Statistics Department* takes a computer-related course, this student could be more eligible. Otherwise, e.g., a sociology student would like to take a mathematics course, there is a chance that the course is out of the student’s comfortable zone.

### Constructing the course enrollment analysis model

For analyzing the cross-college course enrollments, we constructed five different analysis models by the *random forest* algorithm (the code can be found in S2 Code), i.e., Φ_*Baseline*_({*A*′}), Φ_*Average*_({*A*}), Φ_*Hadamard*_({*A*}), Φ_*Weight*−1_({*A*}) and Φ_*Weight*−2_({*A*}), to compare their prediction performances, where {*A*′} and {*A*} are two feature sets consisting of only the logs-based features and all the features presented above. Note that there are four binary operators being used to measure the graph-based features, the corresponding models are named by these operators, and the model trained on only the logs-based features are named as the baseline. From the analysis models, we would obtain the feature importance measurements (FIM) to evaluate the importance of each feature in terms of their impacts on prediction accuracy. For each feature *A*_*i*_, we can rank its importance $\beta_{A_{i}}$ in the corresponding models. Larger *β* value indicates that the feature has a stronger impact on the forecasting task.

*Random forest* is an ensemble learning method for classification, regression and other tasks, which operates by constructing a multitude of decision trees at training stage and outputting the class that is the mode of the classes (classification) or mean prediction (regression) of the individual trees [34]. The *random forest* algorithm has various advantages that make it appropriate to our study [35]. First, the algorithm does not have a linear assumption between the inputs and the outcome, which makes it perform better than the linear-based methods in complex situations. Second, the random sampling mechanism and the out-of-bag estimation approach used in the algorithm make our models less prone to over-fitting. Last but not the least, unlike many machine learning algorithms that work in a black-box style, the *random forest* algorithm allows us to evaluate the importance of each feature, which helps us to further explore and understand the potential causal relations between the inputs and the outcome. Based on the five analysis models, two core questions could be addressed in the experiment section:

- **Can the graph-based features be beneficial to improve analyzing the cross-college course enrollments?**
- **To what extent do the graph-based features contribute to the performances of the analysis models?**

Question 1 addresses the need to validate the performance of the proposed method against the baseline. Specifically, compared with the baseline (i.e., the model constructed via the features presented in Table 1), it needs to examine whether the four contrast models trained on both the logs-based and graph-based features have higher accuracy on the prediction task. If so, we can infer that the information extracted from the contextual graph is conducive to analyzing students’ behaviors on the cross-college course enrollments. And in more general, it is a feasible scheme to transform the variable type from nominal to ratio via the graphical approach, where the latter usually possesses a greater amount of information than the former.

Question 2 focuses on the individual feature level, in which the ranking of each feature is evaluated via the feature importance measurements. In more details, it needs to confirm whether the graph-based features play a more significant role in the prediction task comparing with the log-based features. By investigate this question, we can illustrate what features are the key indicators to analyzing the cross-college course enrollments, which can be potentially helpful to enhance other EDM methodologies.

## Experiments

### Experimental settings

The data used in this study include course enrollment logs of the graduate students at **Indiana University Bloomington** covering 11 academic years from 2006 to 2016. On the whole, this data consist of 24,663 students, 1,674 courses and 417,590 course enrollment records from 12 colleges (86 diplomas) over 34 academic semesters, and the numbers of the various relations are: 417,590 course → student, 24,663 student → college, 1,674 college → course, 24,663 student → diploma and 3,657 student → student respectively. (Additional information about this data can be found in S1 and S2 Tables.)

To be detailed, there are 11,661 female and 13,002 male students, and the age distribution is: 2 students in [0, 20), 17,115 in [20, 30), 6,229 in [30, 40), 1,085 in [40, 50) and 232 in [50, 70). Specifically, 15,500 students pursue for the master’s degree, 8,172 for the doctor’s degree, and the other 991 choose the non-degree programs. Meanwhile, the subject ratio between sciences and arts is 3,473 vs 21,190. On average, the students undertake 16.93 (std = 3.84) courses and earn 49.35 (std = 12.95) credits during their learning careers, and the GPA distribution is: 60 students in (2.0, 2.7], 330 in (2.7, 3.0], 1,649 in (3.0, 3.3], 8,685 in (3.3, 3.7] and 13,939 in (3.7, 4.0]. Each course in this study has an average of 2.91 (std = 0.87) credits, and the students receive 3.76 (std = 0.22) grade points per course on the average. Furthermore, within the 11 academic years, a maximum of 5,022 students enroll in one course altogether, whereas a course requires at least 10 students to start its opening. Comparing with other similar studies, the data employed in this work are significantly larger. By using the method presented in the previous section, a contextual graph is constructed, and then we use the *node2vec* algorithm to obtain the target node/edge vector representations. Next, the graph-based features are extracted following the definitions presented in Table 4.

In this paper, as our main task is to predict students’ behaviors on the cross-college course enrollments, we have filtered out 33,967 cross-college course enrollment records totally (8.13% of all the records), and the related statistics are shown in Table 5. For some administrative reasons, the records in the years 2006 and 2016 are fragmentary, and it can be observed that from the year 2009 on, more than half of the courses are offered to students outside the colleges. It is easy to find that the number of the negative instances (where a student does not enroll a given course outside his/her home college) is much larger than that of the positive ones. To ensure the quality of the analysis models, we first picked up all the positive instances from the whole data, and then performed an *under-sampling* technique (random sampling with replacement) on the negative instances to sample a subset that matches the number of the positive instances [36]. This method has been proofed to be effective in statistics modeling. To be specific, we generated a negative instance by randomly matching a student and a course outside his/her home college. If this matching does not exist in the original data, then we would include it into the negative instance subset. Finally, we would get a training data set containing both 50% the positive instances and 50% the negative ones.

**Table 5:** Statistics for the cross-college course enrollment records.

Year	#Colleges	#Diplomas	#Courses	#Students	#Records
2006	10	18	220	49	271
2007	9	26	589	235	1,508
2008	11	31	779	527	2,557
2009	11	36	1,058	1,338	5,967
2010	10	36	826	815	3,297
2011	11	32	883	748	3,245
2012	11	33	867	769	3,151
2013	11	36	852	765	3,030
2014	12	31	904	769	3,587
2015	12	40	1,130	1,460	6,787
2016	11	21	325	84	567

For balancing the randomness, we have adopted the *easy ensemble* method to sample 10 training data sets for a given analysis model [37], and the result averaged on the 10 runs is taken as the final outcome of the model. As there are five different analysis models studied in this paper, for a fixed parameter setting it needs to sample 50 training data sets in total.

### Experimental results

First of all, in order to assess the usefulness of the graph-based features, we have compared the performances of the five analysis models. Here, the out-of-bag (oob) error estimate is taken as the evaluation criteria, where it is estimated internally during the run and there is no need for cross-validation to get an unbiased estimate of the test error [35]. Meanwhile, as mentioned in the previous section, the effects of the graph-based features rely on two key parameters *p* and *q*, so we have done a *grid search* to tune their values [38]. As recommended, *p*, *q* ∈ {0.25, 0.5, 1, 2, 4}, so there are a total of 25 groups of experiments, each group with 50 (5 × 10) runs (training data sets).

The results of the 25 replications are summarized in Table 6. For the sake of brevity, given an analysis model and a fixed parameter setting, only the value averaged on the 10 runs is listed, and the best value on one group is marked in bold. (The detailed results can be found in S3 Table.) From this table, it can be seen that all the four contrast models trained on both the logs-based and graph-based features are superior to the baseline under all the parameter settings, and the Φ_*Average*_({*A*}) obtains a greater number of the best value than the other models: 0 (Φ_*Baseline*_({*A*′})), 10 (Φ_*Average*_({*A*})), 6 (Φ_*Hadamard*_({*A*})), 8 (Φ_*Weight*−1_({*A*})) and 1 (Φ_*Weight*−2_({*A*})). To be exact, compared to the baseline, the four contrast models gain an average of 11.58% (Φ_*Average*_({*A*})), 11.20% (Φ_*Hadamard*_({*A*})), 11.51% (Φ_*Weight*−1_({*A*})) and 10.95% (Φ_*Weight*−2_({*A*})) decrease on the oob error estimate respectively. Thus, we can draw a preliminary conclusion that the graph-based features do contribute to improving prediction accuracy of the cross-college course enrollments.

**Table 6:** Oob error estimates for the five analysis models.

			out-of-bag error estimate %				
No	*p*	*q*	Baseline	Average	Hadamard	Weight-1	Weight-2
1	0.25	0.25	13.6625	**11.8343**	12.2070	12.0146	12.0457
2	0.25	0.5	13.5950	12.1193	12.1537	**11.6784**	11.7932
3	0.25	1	13.5945	12.1728	12.2288	**11.8020**	11.8466
4	0.25	2	13.6711	12.0916	12.2300	**11.9195**	12.0792
5	0.25	4	13.6747	12.0186	**11.7095**	11.7788	11.8151
6	0.5	0.25	13.6535	**11.9261**	11.9806	11.9437	11.9959
7	0.5	0.5	13.7148	12.2354	**12.1848**	12.2791	12.4092
8	0.5	1	13.6759	12.2741	**12.1923**	12.5525	12.5928
9	0.5	2	13.6067	**11.6952**	11.9264	11.9204	11.9419
10	0.5	4	13.6325	**12.0361**	12.2166	12.1166	12.2647
11	1	0.25	13.6939	**11.8998**	11.9807	12.0293	12.0988
12	1	0.5	13.7525	12.1253	12.1628	**11.8830**	11.9129
13	1	1	13.6778	11.8518	**11.7747**	11.8820	11.9114
14	1	2	13.6351	**12.0707**	12.1829	12.2236	12.2438
15	1	4	13.6104	12.0702	12.2278	**11.9844**	12.1571
16	2	0.25	13.7313	12.4696	12.5539	**12.4171**	12.4695
17	2	0.5	13.6518	12.0691	**12.0130**	12.3602	12.4225
18	2	1	13.6711	**12.1602**	12.2721	12.1787	12.3280
19	2	2	13.6416	**11.9357**	11.9741	11.9532	12.0198
20	2	4	13.6471	12.2640	12.2816	**12.0411**	12.2442
21	4	0.25	13.6705	**12.1806**	12.1924	12.4737	12.5304
22	4	0.5	13.6228	**11.9750**	12.2032	12.1787	12.2883
23	4	1	13.5486	12.2712	12.2958	12.0888	**12.0805**
24	4	2	13.6560	12.1593	12.2252	**12.1360**	12.2172
25	4	4	13.6007	11.8684	**11.7046**	12.1745	12.2042
Mean			13.6517	**12.0710**	12.1230	12.0804	12.1565
Std			0.0445	0.1675	0.1765	0.2203	0.2256

For investigating the influence of the parameters *p* and *q* on the four contrast models, we have calculated the significance ranking of each parameter as in Table 7. From this table, it can be observed that compared to the *q*, the four models are much more sensitive to the *p*, especially for the Φ_*Weight*−1_({*A*}) and Φ_*Weight*−2_({*A*}). In the *node2vec*, the parameter *p* controls the possibility of directly revisiting a node in the biased random walk, where setting it to a low value (<*min*(*q*, 1)) would encourage the walk to backtrack a move and hold the walk quite close to the starting node, as shown in Eq 3. And it is easy to find that for the four contrast models, the best value of the *p* is lower than or equal to 1, whereas that of the *q* is higher than or equal to 1. In this circumstance, the biased random walk would obtain a local view of the contextual graph, which means that the emphasis of the graph would be placed on the structural equivalence, and the nodes that have the similar structural roles in the graph should be embedded closely together. This is because that, the structural equivalence based on the graph roles such as bridges and hubs, can be inferred by just observing the immediate neighbors of each node [14]. As mentioned above, we still take *Data Structure* and *C Programming* as an example, owing to quite a number of students having taken both the courses, the two courses would play very similar structural roles (the hub) in the two distinct student communities. Therefore, if the vector representations for the two courses could resemble each other, then for a student who took (was close to) one of them, he/she could have a relatively high probability to take the other one.

**Table 7:** Significance ranking of each parameter.

	*p*				*q*			
Value	Average	Hadamard	Weight-1	Weight-2	Average	Hadamard	Weight-1	Weight-2
0.25	12.0473	12.1058	**11.8387**	**11.9160**	12.0621	12.1829	12.1757	12.2280
0.5	12.0334	12.1001	12.1625	12.2409	12.1048	12.1435	12.0759	12.1652
1	**12.0036**	**12.0658**	12.0005	12.0648	12.1460	12.1527	12.1008	12.1519
2	12.1797	12.2189	12.1901	12.2968	**11.9905**	12.1077	12.0306	**12.1004**
4	12.0909	12.1242	12.2104	12.2641	12.0515	**12.0280**	**12.0191**	12.1371
Min	12.0036	12.0658	11.8387	11.9160	11.9905	12.0280	12.0191	12.1004
Max	12.1797	12.2189	12.2104	12.2968	12.1460	12.1829	12.1757	12.2280
Delta	0.1761	0.1532	0.3717	0.3808	0.1555	0.1549	0.1566	0.1277
Ranking	3	7	2	1	5	6	4	8

**Fig 6:**
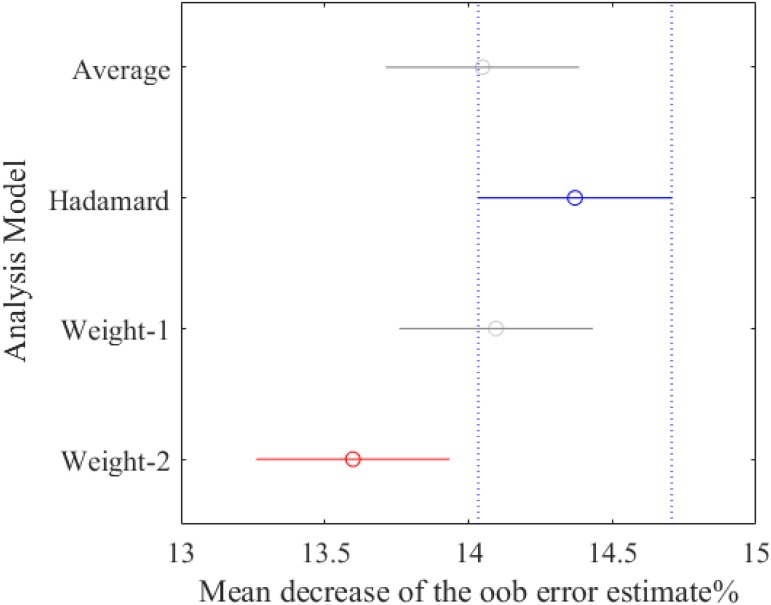
Multiple comparison of decreases on the oob error estimates for the three contrast models.

In order to better verify the effects of the graph-based features on the prediction task, firstly we have selected the best parameter settings for the four contrast models, and then ranked the FIMs of all the features. As shown in Table 7, the best parameter settings for the four models are as follows: *p* = 0.5, *q* = 2 (Φ_*Average*_({*A*})); *p* = 0.25, *q* = 4 (Φ_*Hadamard*_({*A*})); *p* = 0.25, *q* = 0.5 (Φ_*Weight*−1_({*A*})); and *p* = 0.25, *q* = 0.5 (Φ_*Weight*−2_({*A*})). To make it fair, we have calculated their decreases on the oob error estimates when compared to the corresponding baseline, and the statistical differences among them are indicated through the *one-way ANOVA* (95% confidence). Fig 6 illustrates the corresponding multiple comparison. From this figure, it can be seen that under the best parameter setting, the performances of the first three models are quite similar to each other (with the confidence intervals overlapping), while the performance of the Φ_*Hadamard*_({*A*}) is significantly better than that of the Φ_*Weight*−2_({*A*}). In this manner, only the first three contrast models will be used in the following FIM analysis, and we have collected the FIMs of all the features from the three models on the 10 runs, as shown in S4 Table.

**Fig 7:**
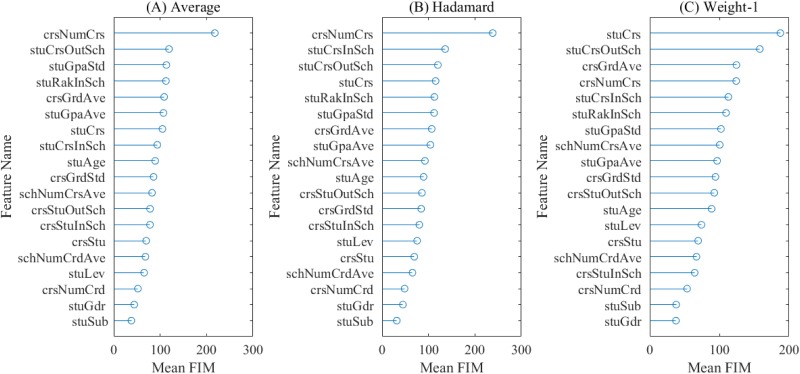
Mean FIMs for the three contrast models. (A) Average. (B) Hadamard. (C) Weight-1.

Fig 7 graphically presents the FIMs averaged on the 10 runs for the first three contrast models. According to this figure, it can be found that three graph-based features, i.e., *stuCrs*, *stuCrsInSch* and *stuCrsOutSch* (the course preference) dominate the top of the three mean FIM lists, even if their rankings among the three models are not exactly the same. And their rankings in the Φ_*Weight*−1_({*A*}) are higher than those in the other two, where *stuCrs*, *stuCrsOutSch* and *stuCrsInSch* rank the 1*^st^*, 2*^nd^* and 5*^th^* respectively. As for the remaining three graph-based features (the course appropriateness), their rankings are lower than average in all the three mean FIM lists, which means that they have relatively limited effects on predicting the cross-college course enrollments. In general, the course preference is much more important than the course appropriateness when analyzing whether a student would enroll a course outside his/her home college.

**Fig 8:**
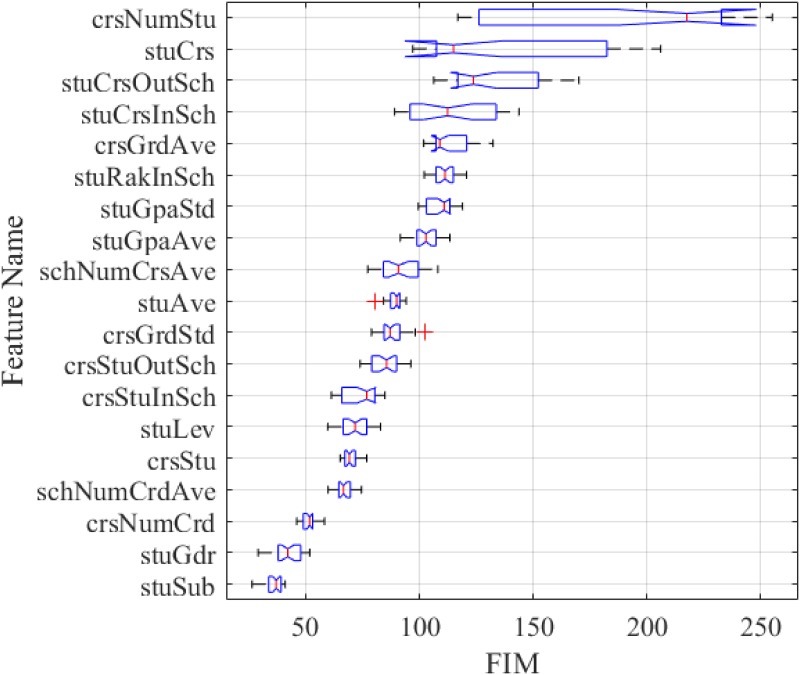
Box plots of FIMs for all the features.

**Fig 9:**
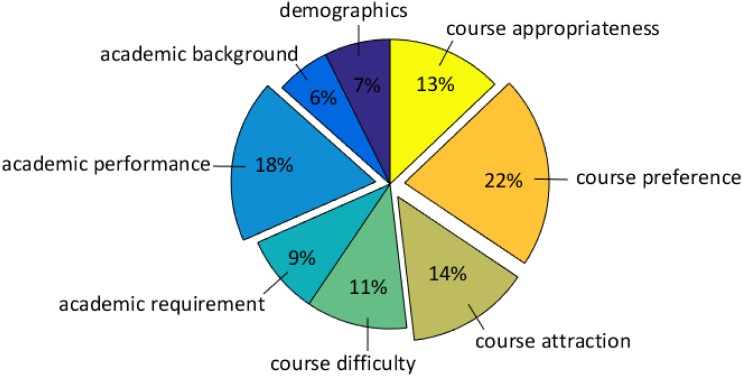
Pie chart of FIMs on the feature categories.

Furthermore, we have conducted an overall comparison of the FIMs for all the features, and it should be noted that our conclusion is based on all the results from S4 Table (not the mean). The box plots of the FIMs for all the features are given in Fig 8, where the features are sorted by the mean FIM in a descending order. From this figure, it can be seen that the course preference occupies the positions from the 2*^nd^* to 4*^th^*, while for the course appropriateness, its features rank the 12*^th^*, 13*^th^* and 15*^th^* respectively. Then we have added up the mean FIM of each feature according to the feature category, and the corresponding pie chart is presented in Fig 9. As can be seen from this figure that the top three important feature categories are: the course preference, the academic performance and the course attraction, followed by the course appropriateness, the course difficulty, the academic requirement, the demographics and the academic background. Despite that the features belonging to the course appropriateness do not have relatively high FIMs individually, the combination of them stays at the 4*^th^* among all the categories. Hence, we can conclude that the two groups of graph-based features have a marked impact on the prediction task, and the course preference is the most important of all. Nevertheless, the sizes of box plots of the top four features are remarkably larger than the others, demonstrating that they have a high degree of disagreements among the three contrast models.

For the sake of giving an exact order of the FIMs for all the features, the *Friedman test*, a non-parametric statistical method is introduced to obtain the precise rankings for them. Through sorting the FIMs in S4 Table in a descending way row by row, we can get the ranking of each FIM on each row. After that, we can average the rankings for all the features on the whole, as listed in Table 8. From this table, it can be observed that the orders of features are almost the same as those in Fig 8, which means that the conclusions drawn by Figs 8 and 9 are sustained. Although for the course preference, its corresponding features have high variance, at 95% confidence level, they still occupy the positions from the 2*^nd^* to 4*^th^*. And except for *crsNumStu*, the three features in the course preference have higher rankings than the rest. In summary, we can infer that the information extracted from the contextual graph do play a key role in analyzing students’ behaviors on the cross-college course enrollments, especially for the three features belonging to the course preference.

**Table 8:** Friedman rankings for all the features.

No	Feature Name	Ranking	Category
1	crsNumStu	1.3000	
2	stuCrsOutSch	1.7667	
3	stuCrs	2.9333	
4	stuCrsInSch	4.8667	
5	stuRakInSch	5.4667	
6	crsGrdAve	5.6667	
7	stuGpaStd	6.3667	
8	stuGpaAve	8.7667	
9	schNumCrsAve	9.2333	
10	stuAge	9.8333	
11	crsGrdStd	10.5000	
12	crsStuOutSch	12.6668	
13	crsStuInSch	14.6333	
14	stuLev	14.6667	
15	crsStu	14.8333	
16	schNumCrdAve	14.8333	
17	crsNumCrd	16.1333	
18	stuGdr	17.3667	
19	stuSub	18.1667	

▭ demographics ▭ academic background ▭ academic performance ▭ academic requirement ▭ course difficulty ▭ course attraction ▭ course preference ▭ course appropriateness

**Fig 10:**
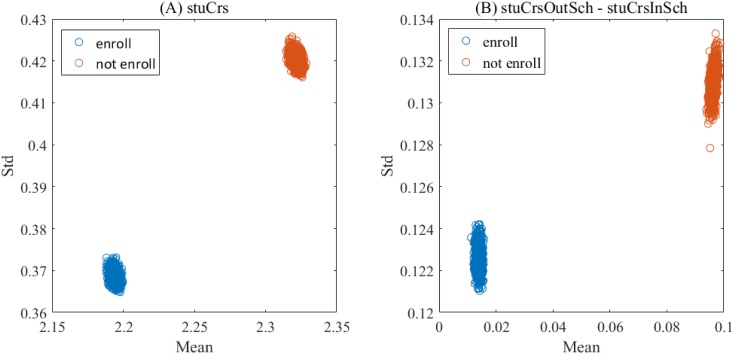
Value distributions of features in the course preference. (A) stuCrs. (B) stuCrsOutSch-stuCrsInSch.

Moreover, since the course preference is the most major factor of the analysis models, we are to display the value distributions of its corresponding features in terms of whether a student would enroll a course outside his/her home college. As the training data sets in the experiment contain only a small fraction of the whole data space, we have adopted the *bootstrap sampling* (1000 samples) to gauge the mean and standard error of these features. Taking Φ_*Weight*−1_({*A*}) as an example, Fig 10 graphically illustrates the mean and standard error of the three features according to enrollment status. From this figure, it can be seen that for the students who would enroll a given course outside his/her home college, no matter the mean or standard error of *stuCrs* is distinctly lower than that for the ones who would not, and no overlap exists between the two groups of students. Similar observations can be made in *stuCrsOutSch-stuCrsInSch*, indicating that the enrollment decisions are tightly linked to the value distributions of the features in the course preference. In other words, the three graph-based features can be an essential indicator to measure the student’s interest in a course, and are useful to predicting the course enrollments, which is consistent to the previous studies [30, 31].

## Conclusion

In this paper, we have proposed a novel method for exploring students’ behaviors on the cross-college course enrollments from the perspective of graph mining. The framework of the proposed method provides an effective mechanism to transform the initially isolated variables (the nominal) to the interrelated ones (the ratio), where the implicit information hidden in the data becomes measurable quantitatively. For this method, a contextual graph is constructed in the light of various organizational relations within the course enrollment logs, and the *node2vec* algorithm is employed to characterize the vector representations of nodes and edges on the graph. By leveraging the graph-based features generated from the contextual graph, four *random forest* classifiers are implemented as analysis models to infer whether a student would enroll a given course outside his/her home college in the coming semester.

Experiments on 24,663 students, 1,674 courses and 417,590 enrollment records demonstrate that these graph-based features can successfully improve analyzing the cross-college course enrollments, in which *stuCrs*, *stuCrsInSch* and *stuCrsOutSch* significantly outperform most of the other features. This finding proves that the student’s course preference plays a pivotal role in deciding future course enrollments, which means that the previous conclusion that regards the course interest as a critical factor is sustained. Meanwhile, we have also investigated if these three new features are statistically important to characterize a student’s course preference, and the value distributions indicate a close association between them and enrollment decisions. Besides, when the contextual graph exhibits more about the structural equivalence, the corresponding graph-based features would have a better performance on the prediction task.

Although the selected graph-based features in this study are all focusing on the distance between a student and a course, the proposed method enables the distance calculation between any pair of nodes in the contextual graph. For example, we can measure distances among a number of courses such as *HyperText Markup Language*, *Web programming* and *Database*, and this kind of information is able to offer reference for administrators to formulate a new syllabus or learning program. Another example are distances between institutions, like *Informatics College* and *Library Science School*, as computer applications are growing popularity gradually, the distance could be narrowing year after year, which can aid decision making or explain reasons to the faculty adjustment. Furthermore, the proposed method permits additional organizational relations beyond those listed in Table 2. But in this case, the transition probability in the contextual graph needs to be re-scaled because some edge types would have very different weights, and how to tune these weights will also be one of our next studies.

Our future work will cover four main areas. Firstly, limited by available data, only nineteen features are considered in this study, a potential future direction is to take more sophisticated features into account, such as the subjective rating data collected by questionnaires, interviews or web interfaces [22]. Secondly, as the enrollment decisions can be influenced by various kinds of social factors like friend recommendations, we would like to incorporate the positions and communities of students in the social networks to ameliorate our analysis [5, 39]. Meanwhile, the relations between various courses are ignored as well, which could be resolved by means of the global knowledge graph data in the future. Thirdly, by leveraging the outcomes from this paper, we are going to investigate other kinds of machine learning algorithms apart from the *random forest* for the cross-college course enrollments. Finally, as it is feasible to transform the variable type via a contextual graph, we are going to apply and generalize the proposed method to some other statistical analysis problems (e.g., transforming nominal variables to ratio variables by using graph mining).

## Supporting information

S1 Code Python code of the node2vec algorithm. https://github.com/snap-stanford/snap/tree/master/examples/node2vec.
(ZIP)

S2 Code Fortran code of the random forest algorithm. https://www.stat.berkeley.edu/~breiman/RandomForests/cc_software.htm.
(ZIP)

S1 Table Course titles of this study. (CSV)

S2 Table Course statistics of this study. (CSV)

S3 Table Detailed results for the 25 groups of experiments. (XLSX)

S4 Table FIMs of features from three contrast models on the 10 runs of experiments. (XLSX)
